# Vulnerability and Resilience Among Older Adults During the COVID Pandemic

**DOI:** 10.1093/geroni/igab046.500

**Published:** 2021-12-17

**Authors:** Carolyn Aldwin

## Abstract

The COVID-19 pandemic is particularly challenging for many older adults. They are strongly encouraged to practice social distancing and sheltering in place to decrease morbidity and mortality risks. However, social isolation and loneliness increase the risk of mental and physical health problems. Nonetheless, there are indications that older adults may be more resilient than originally thought. Park et al. present longitudinal findings that younger adults fared worse than middle-aged or older ones, reporting greater distress and less social support, mindfulness, and emotion regulation skills. For older participants, acceptance of negative emotions and social support predicted lower distress. Choun et al. also present longitudinal data, showing that depressive symptoms decreased among older adults during the lockdown phase of the pandemic, but reflected changes in loneliness and physical health symptoms. Stellman et al. found that moderate levels of combat experience were helpful for some older Vietnam veterans coping with the pandemic, although a few found that it made coping more difficult. Turner et al. found that positive, non-familial intergenerational contacts were associated with a higher number of positive pandemic-related changes, such as finding new hobbies and meaning in work. Finally, Aldwin et al. present a social ecological approach to vulnerability and resilience. Using qualitative data, they found that stressors and resources were reported at personal, interpersonal and societal levels. Further, older adults both took comfort from and contributed to community resources during this pandemic. In summary, this symposium identified factors that contribute to older adults’ resilience during this pandemic.

